# *exo*-4-[(1*H*-Benzimidazol-2-yl)meth­yl]-10-oxa-4-aza­tricyclo­[5.2.1.0^2,6^]decane-3,5-dione

**DOI:** 10.1107/S1600536811026602

**Published:** 2011-07-09

**Authors:** Shi-Kun Li, Fan Zhang, Tian-Xi Lv, Qiu-Yue Lin

**Affiliations:** a Zhejiang Key Laboratory for Reactive Chemistry on Solid Surfaces, Institute of Physical Chemistry, Zhejiang Normal University, Jinhua 321004, Zhejiang, People’s Republic of China; b College of Chemistry and Life Science, Zhejiang Normal University, Jinhua 321004, Zhejiang, People’s Republic of China

## Abstract

In the title compound, C_16_H_15_N_3_O_3_, the dihedral angle between the approximately planar benzimidazolyl group (r.m.s. deviation = 0.010 Å) and the pyrrolidine ring is 78.20 (6)°. The C—C—N bond angle of the bridging CH_2_ group is 112.14 (16)°. In the crystal, mol­ecules are linked *via* N—H⋯N hydrogen bonds, forming infinite chains parallel to [101] and [10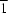].

## Related literature

For the bioactivity of norcantharidin (systematic name 4,10-dioxatricyclo­[5.2.1.0^2,6^]decane-3,5-dione), see: Wang (1989 ▶). For the use of norcantharidin in synthesis, see: Hill *et al.* (2007 ▶). For a related structure, see: Zhu & Lin (2009 ▶).
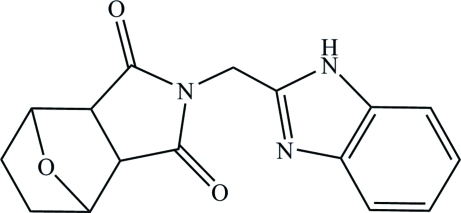


## Experimental

### 

#### Crystal data

C_16_H_15_N_3_O_3_
*M* *_r_* = 297.31
Orthorhombic, 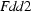

*a* = 17.4294 (2) Å
*b* = 48.2746 (6) Å
*c* = 6.7947 (1) Å
*V* = 5717.04 (13) Å^3^
*Z* = 16
Mo *K*α radiation
μ = 0.10 mm^−1^
*T* = 296 K
0.24 × 0.17 × 0.10 mm

#### Data collection

Bruker SMART APEXII CCD diffractometer
Absorption correction: multi-scan (*SADABS*; Sheldrick, 1996 ▶) *T*_min_ = 0.981, *T*_max_ = 0.990
22741 measured reflections
2159 independent reflections
1690 reflections with *I* > 2σ(*I*)
*R*_int_ = 0.038

#### Refinement

*R*[*F* ^2^ > 2σ(*F* ^2^)] = 0.035
*wR*(*F* ^2^) = 0.087
*S* = 1.04
2159 reflections
199 parameters
1 restraint
H-atom parameters constrained
Δρ_max_ = 0.10 e Å^−3^
Δρ_min_ = −0.17 e Å^−3^

### 

Data collection: *APEX2* (Bruker, 2006 ▶); cell refinement: *SAINT* (Bruker, 2006 ▶); data reduction: *SAINT*; program(s) used to solve structure: *SHELXS97* (Sheldrick, 2008 ▶); program(s) used to refine structure: *SHELXL97* (Sheldrick, 2008 ▶); molecular graphics: *SHELXTL* (Sheldrick, 2008 ▶); software used to prepare material for publication: *SHELXL97*.

## Supplementary Material

Crystal structure: contains datablock(s) I, global. DOI: 10.1107/S1600536811026602/qk2008sup1.cif

Structure factors: contains datablock(s) I. DOI: 10.1107/S1600536811026602/qk2008Isup2.hkl

Supplementary material file. DOI: 10.1107/S1600536811026602/qk2008Isup3.cml

Additional supplementary materials:  crystallographic information; 3D view; checkCIF report

## Figures and Tables

**Table 1:** Hydrogen-bond geometry (Å, °)

*D*—H⋯*A*	*D*—H	H⋯*A*	*D*⋯*A*	*D*—H⋯*A*
N1—H1*A*⋯N2^i^	0.86	1.97	2.827 (2)	175

Symmetry code: (i) 
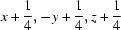
.

**Fig. 1.**
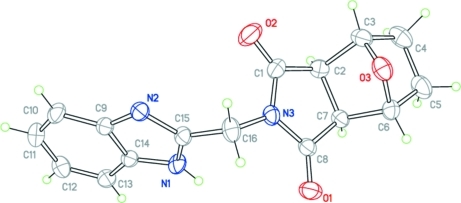
A view of the molecule of (I) showing the atom-labelling scheme with displacement ellipsoids drawn at 30% probability.

## supplementary crystallographic information

### Comment

Norcantharidin is an important compound that has a variety of bioactivities, such as protein kinase inhibition and antitumor activity (Wang, 1989). As a contribution to structure-activity studies in this field, the title compound, (I), a norcantharidin imide (Hill *et al.*, 2007), was synthesized and investigated by X-ray crystallography. A related norcantharidin imide was reported by Zhu & Lin (2009).

X-ray crystallography confirmed the anticipated molecular structure and the atom connectivity for the title compound, as illustrated in Fig. 1. The norcantharidin imide and the benzimidazole moiety of (I) are rigid and feature usual bond lengths and angles. The dihedral angle between the flat benzimidazolyl group (C9—C15,N1,N2) and the pyrrolidine ring (C1,C2,C7,C8,N3) is 78.20 (6)°. The bond angle of the bridging CH_2_ group is C15—C16—N3 = 112.1 (2)°. In the crystal structure the molecules are linked *via* the hydrogen bond N1—H1A···N2^i^, N1···N2^i^ = 2.827 (2) Å ((i): x+1/4, -y+1/4, z+1/4), to form infinite hydrogen bonded chains parallel to [101] and [101].

### Experimental

A mixture of 0.5 mmol norcantharidin, 0.5 mmol 2-(aminomethyl)benzimidazole, 0.5 mmol cadmium acetate as a promoter, and 10 mL distilled water was sealed in a 25 mL Teflon-lined stainless vessel and heated at 433 K for 3 d, then cooled slowly to room temperature. The solution was filtered and gave then block-like transparent crystals.

### Refinement

The structure was solved by direct methods and successive Fourier difference synthesis. The H atoms were positioned geometrically and refined using a riding model [aromatic C—H = 0.93 Å, aliphatic C—H = 0.97Å, and N—H = 0.86 Å, *U*_iso_(H) = 1.2*U*_eq_(C, N)]. In the absence of significant anomalous scattering, the Friedel pairs of the polar structure were merged.

### Crystal data

**Table:**

C_16_H_15_N_3_O_3_	*F*(000) = 2496
*M**_r_* = 297.31	*D*_x_ = 1.382 Mg m^−^^3^
Orthorhombic, *F**d**d*2	Mo *K*α radiation, λ = 0.71073 Å
Hall symbol: F 2 -2d	Cell parameters from 5637 reflections
*a* = 17.4294 (2) Å	θ = 2.5–29.8°
*b* = 48.2746 (6) Å	µ = 0.10 mm^−^^1^
*c* = 6.7947 (1) Å	*T* = 296 K
*V* = 5717.04 (13) Å^3^	Block, colourless
*Z* = 16	0.24 × 0.17 × 0.10 mm

### Data collection

**Table:**

Bruker SMART APEXII CCD diffractometer	2159 independent reflections
Radiation source: fine-focus sealed tube	1690 reflections with *I* > 2σ(*I*)
graphite	*R*_int_ = 0.038
ω scans	θ_max_ = 29.8°, θ_min_ = 2.5°
Absorption correction: multi-scan (*SADABS*; Sheldrick, 1996)	*h* = −24→24
*T*_min_ = 0.981, *T*_max_ = 0.990	*k* = −67→66
22741 measured reflections	*l* = −9→8

### Refinement

**Table:**

Refinement on *F*^2^	Secondary atom site location: difference Fourier map
Least-squares matrix: full	Hydrogen site location: inferred from neighbouring sites
*R*[*F*^2^ > 2σ(*F*^2^)] = 0.035	H-atom parameters constrained
*wR*(*F*^2^) = 0.087	*w* = 1/[σ^2^(*F*_o_^2^) + (0.0448*P*)^2^ + 1.1097*P*] where *P* = (*F*_o_^2^ + 2*F*_c_^2^)/3
*S* = 1.04	(Δ/σ)_max_ < 0.001
2159 reflections	Δρ_max_ = 0.10 e Å^−^^3^
199 parameters	Δρ_min_ = −0.17 e Å^−^^3^
1 restraint	Absolute structure: 1753 Friedel pairs were merged
Primary atom site location: structure-invariant direct methods	

### Special details

**Table:**

Geometry. All e.s.d.'s (except the e.s.d. in the dihedral angle between two l.s. planes) are estimated using the full covariance matrix. The cell e.s.d.'s are taken into account individually in the estimation of e.s.d.'s in distances, angles and torsion angles; correlations between e.s.d.'s in cell parameters are only used when they are defined by crystal symmetry. An approximate (isotropic) treatment of cell e.s.d.'s is used for estimating e.s.d.'s involving l.s. planes.
Refinement. Refinement of *F*^2^ against ALL reflections. The weighted *R*-factor *wR* and goodness of fit *S* are based on *F*^2^, conventional *R*-factors *R* are based on *F*, with *F* set to zero for negative *F*^2^. The threshold expression of *F*^2^ > σ(*F*^2^) is used only for calculating *R*-factors(gt) *etc*. and is not relevant to the choice of reflections for refinement. *R*-factors based on *F*^2^ are statistically about twice as large as those based on *F*, and *R*- factors based on ALL data will be even larger.

### Fractional atomic coordinates and isotropic or equivalent isotropic displacement parameters (Å^2^)

**Table:**

	*x*	*y*	*z*	*U*_iso_*/*U*_eq_	
N2	−0.06276 (8)	0.10833 (3)	0.2792 (3)	0.0454 (4)	
N1	0.04528 (8)	0.11596 (3)	0.4450 (3)	0.0428 (4)	
H1A	0.0893	0.1229	0.4740	0.051*	
N3	−0.02809 (8)	0.16452 (3)	0.1037 (3)	0.0431 (4)	
O2	−0.07386 (9)	0.14915 (3)	−0.1930 (3)	0.0629 (4)	
O1	−0.00312 (11)	0.18963 (4)	0.3811 (3)	0.0765 (5)	
O3	−0.02560 (7)	0.22474 (3)	−0.0973 (2)	0.0556 (4)	
C1	−0.07375 (10)	0.16595 (3)	−0.0624 (3)	0.0439 (4)	
C2	−0.12089 (10)	0.19215 (3)	−0.0513 (3)	0.0431 (4)	
H2B	−0.1761	0.1887	−0.0634	0.052*	
C3	−0.09207 (12)	0.21403 (4)	−0.1970 (3)	0.0527 (5)	
H3A	−0.0813	0.2068	−0.3288	0.063*	
C4	−0.14630 (14)	0.23883 (4)	−0.1956 (5)	0.0713 (7)	
H4A	−0.1995	0.2330	−0.2016	0.086*	
H4B	−0.1357	0.2513	−0.3041	0.086*	
C5	−0.12741 (14)	0.25219 (4)	0.0024 (4)	0.0700 (7)	
H5A	−0.1088	0.2710	−0.0139	0.084*	
H5B	−0.1716	0.2523	0.0892	0.084*	
C6	−0.06469 (12)	0.23307 (4)	0.0784 (4)	0.0552 (5)	
H6A	−0.0311	0.2417	0.1763	0.066*	
C7	−0.09930 (11)	0.20533 (4)	0.1463 (3)	0.0464 (5)	
H7A	−0.1434	0.2077	0.2341	0.056*	
C8	−0.03878 (12)	0.18644 (4)	0.2306 (3)	0.0492 (5)	
C16	0.02831 (10)	0.14281 (4)	0.1375 (4)	0.0519 (5)	
H16A	0.0760	0.1511	0.1814	0.062*	
H16B	0.0384	0.1333	0.0145	0.062*	
C15	0.00198 (10)	0.12236 (3)	0.2871 (3)	0.0410 (4)	
C9	−0.06167 (9)	0.09153 (3)	0.4452 (3)	0.0391 (4)	
C10	−0.11496 (11)	0.07221 (4)	0.5106 (3)	0.0488 (5)	
H10A	−0.1597	0.0687	0.4402	0.059*	
C11	−0.09925 (12)	0.05847 (4)	0.6828 (4)	0.0571 (5)	
H11A	−0.1339	0.0453	0.7288	0.068*	
C12	−0.03280 (13)	0.06372 (5)	0.7903 (4)	0.0600 (6)	
H12A	−0.0247	0.0543	0.9078	0.072*	
C13	0.02144 (11)	0.08263 (4)	0.7270 (3)	0.0526 (5)	
H13A	0.0660	0.0861	0.7984	0.063*	
C14	0.00597 (10)	0.09617 (3)	0.5518 (3)	0.0395 (4)	

### Atomic displacement parameters (Å^2^)

**Table:**

	*U*^11^	*U*^22^	*U*^33^	*U*^12^	*U*^13^	*U*^23^
N2	0.0332 (7)	0.0520 (8)	0.0510 (10)	−0.0018 (6)	−0.0111 (7)	0.0068 (8)
N1	0.0308 (7)	0.0445 (7)	0.0532 (10)	−0.0010 (6)	−0.0124 (8)	−0.0003 (7)
N3	0.0394 (8)	0.0408 (7)	0.0490 (10)	0.0022 (6)	−0.0058 (7)	0.0065 (7)
O2	0.0768 (10)	0.0467 (7)	0.0652 (11)	0.0086 (7)	−0.0172 (9)	−0.0142 (8)
O1	0.0876 (11)	0.0950 (13)	0.0468 (11)	0.0081 (9)	−0.0214 (10)	−0.0072 (9)
O3	0.0472 (7)	0.0489 (7)	0.0708 (11)	−0.0026 (6)	0.0051 (8)	0.0100 (7)
C1	0.0451 (9)	0.0351 (8)	0.0514 (12)	−0.0021 (7)	−0.0074 (9)	0.0015 (8)
C2	0.0380 (9)	0.0387 (8)	0.0525 (11)	0.0014 (7)	−0.0062 (9)	−0.0002 (8)
C3	0.0624 (12)	0.0469 (10)	0.0489 (13)	0.0087 (9)	−0.0070 (11)	0.0047 (9)
C4	0.0740 (15)	0.0457 (10)	0.094 (2)	0.0098 (10)	−0.0194 (15)	0.0099 (13)
C5	0.0703 (13)	0.0407 (9)	0.099 (2)	0.0100 (10)	0.0017 (15)	−0.0043 (12)
C6	0.0576 (12)	0.0427 (9)	0.0654 (15)	−0.0041 (8)	−0.0085 (11)	−0.0086 (10)
C7	0.0447 (10)	0.0481 (9)	0.0466 (12)	0.0029 (8)	0.0035 (9)	−0.0034 (9)
C8	0.0493 (11)	0.0568 (11)	0.0414 (12)	−0.0011 (9)	−0.0015 (10)	0.0022 (9)
C16	0.0386 (9)	0.0511 (10)	0.0660 (15)	0.0073 (8)	−0.0002 (10)	0.0147 (10)
C15	0.0324 (8)	0.0416 (8)	0.0490 (12)	0.0040 (7)	−0.0080 (8)	0.0041 (8)
C9	0.0334 (8)	0.0393 (8)	0.0444 (11)	0.0033 (6)	−0.0079 (8)	−0.0022 (8)
C10	0.0394 (9)	0.0502 (10)	0.0570 (14)	−0.0041 (8)	−0.0060 (9)	−0.0017 (9)
C11	0.0535 (11)	0.0506 (10)	0.0671 (15)	−0.0001 (8)	0.0027 (12)	0.0114 (11)
C12	0.0610 (12)	0.0633 (12)	0.0558 (14)	0.0089 (10)	−0.0030 (11)	0.0175 (11)
C13	0.0470 (11)	0.0628 (11)	0.0480 (12)	0.0076 (9)	−0.0156 (10)	0.0025 (10)
C14	0.0333 (8)	0.0388 (8)	0.0463 (12)	0.0042 (7)	−0.0079 (8)	−0.0021 (8)

### Geometric parameters (Å, °)

**Table:**

N2—C15	1.317 (2)	C5—C6	1.521 (3)
N2—C9	1.389 (2)	C5—H5A	0.9700
N1—C15	1.348 (3)	C5—H5B	0.9700
N1—C14	1.381 (2)	C6—C7	1.540 (3)
N1—H1A	0.8600	C6—H6A	0.9800
N3—C8	1.378 (3)	C7—C8	1.507 (3)
N3—C1	1.383 (3)	C7—H7A	0.9800
N3—C16	1.455 (2)	C16—C15	1.490 (3)
O2—C1	1.202 (2)	C16—H16A	0.9700
O1—C8	1.206 (3)	C16—H16B	0.9700
O3—C6	1.432 (3)	C9—C10	1.389 (3)
O3—C3	1.438 (3)	C9—C14	1.402 (2)
C1—C2	1.510 (2)	C10—C11	1.373 (3)
C2—C3	1.532 (3)	C10—H10A	0.9300
C2—C7	1.533 (3)	C11—C12	1.393 (3)
C2—H2B	0.9800	C11—H11A	0.9300
C3—C4	1.525 (3)	C12—C13	1.383 (3)
C3—H3A	0.9800	C12—H12A	0.9300
C4—C5	1.528 (4)	C13—C14	1.385 (3)
C4—H4A	0.9700	C13—H13A	0.9300
C4—H4B	0.9700		
			
C15—N2—C9	104.80 (15)	C5—C6—H6A	113.7
C15—N1—C14	107.39 (14)	C7—C6—H6A	113.7
C15—N1—H1A	126.3	C8—C7—C2	104.70 (15)
C14—N1—H1A	126.3	C8—C7—C6	111.46 (16)
C8—N3—C1	113.23 (15)	C2—C7—C6	101.23 (17)
C8—N3—C16	123.08 (17)	C8—C7—H7A	112.9
C1—N3—C16	123.63 (17)	C2—C7—H7A	112.9
C6—O3—C3	96.35 (15)	C6—C7—H7A	112.9
O2—C1—N3	124.71 (16)	O1—C8—N3	124.0 (2)
O2—C1—C2	126.96 (19)	O1—C8—C7	127.3 (2)
N3—C1—C2	108.33 (16)	N3—C8—C7	108.75 (17)
C1—C2—C3	111.50 (17)	N3—C16—C15	112.14 (16)
C1—C2—C7	104.94 (16)	N3—C16—H16A	109.2
C3—C2—C7	101.50 (14)	C15—C16—H16A	109.2
C1—C2—H2B	112.7	N3—C16—H16B	109.2
C3—C2—H2B	112.7	C15—C16—H16B	109.2
C7—C2—H2B	112.7	H16A—C16—H16B	107.9
O3—C3—C4	102.38 (16)	N2—C15—N1	113.22 (17)
O3—C3—C2	101.96 (16)	N2—C15—C16	125.23 (18)
C4—C3—C2	109.49 (19)	N1—C15—C16	121.52 (16)
O3—C3—H3A	113.9	C10—C9—N2	129.97 (16)
C4—C3—H3A	113.9	C10—C9—C14	120.28 (17)
C2—C3—H3A	113.9	N2—C9—C14	109.74 (15)
C3—C4—C5	101.72 (19)	C11—C10—C9	117.63 (18)
C3—C4—H4A	111.4	C11—C10—H10A	121.2
C5—C4—H4A	111.4	C9—C10—H10A	121.2
C3—C4—H4B	111.4	C10—C11—C12	121.7 (2)
C5—C4—H4B	111.4	C10—C11—H11A	119.2
H4A—C4—H4B	109.3	C12—C11—H11A	119.2
C6—C5—C4	101.42 (18)	C13—C12—C11	121.7 (2)
C6—C5—H5A	111.5	C13—C12—H12A	119.1
C4—C5—H5A	111.5	C11—C12—H12A	119.1
C6—C5—H5B	111.5	C12—C13—C14	116.48 (19)
C4—C5—H5B	111.5	C12—C13—H13A	121.8
H5A—C5—H5B	109.3	C14—C13—H13A	121.8
O3—C6—C5	103.2 (2)	N1—C14—C13	132.96 (17)
O3—C6—C7	101.06 (15)	N1—C14—C9	104.84 (16)
C5—C6—C7	110.35 (16)	C13—C14—C9	122.19 (18)
O3—C6—H6A	113.7		
			
C8—N3—C1—O2	179.8 (2)	C16—N3—C8—O1	−0.5 (3)
C16—N3—C1—O2	2.6 (3)	C1—N3—C8—C7	0.7 (2)
C8—N3—C1—C2	0.9 (2)	C16—N3—C8—C7	177.93 (16)
C16—N3—C1—C2	−176.28 (16)	C2—C7—C8—O1	176.4 (2)
O2—C1—C2—C3	−71.9 (2)	C6—C7—C8—O1	67.8 (3)
N3—C1—C2—C3	106.99 (19)	C2—C7—C8—N3	−2.0 (2)
O2—C1—C2—C7	179.06 (19)	C6—C7—C8—N3	−110.60 (19)
N3—C1—C2—C7	−2.10 (19)	C8—N3—C16—C15	77.7 (2)
C6—O3—C3—C4	55.9 (2)	C1—N3—C16—C15	−105.4 (2)
C6—O3—C3—C2	−57.41 (16)	C9—N2—C15—N1	0.1 (2)
C1—C2—C3—O3	−77.96 (19)	C9—N2—C15—C16	178.43 (18)
C7—C2—C3—O3	33.32 (17)	C14—N1—C15—N2	−0.4 (2)
C1—C2—C3—C4	174.13 (18)	C14—N1—C15—C16	−178.74 (17)
C7—C2—C3—C4	−74.6 (2)	N3—C16—C15—N2	55.2 (3)
O3—C3—C4—C5	−35.0 (2)	N3—C16—C15—N1	−126.70 (19)
C2—C3—C4—C5	72.7 (2)	C15—N2—C9—C10	−179.28 (19)
C3—C4—C5—C6	1.0 (2)	C15—N2—C9—C14	0.14 (19)
C3—O3—C6—C5	−55.61 (16)	N2—C9—C10—C11	−179.60 (19)
C3—O3—C6—C7	58.61 (15)	C14—C9—C10—C11	1.0 (3)
C4—C5—C6—O3	33.5 (2)	C9—C10—C11—C12	0.6 (3)
C4—C5—C6—C7	−73.8 (2)	C10—C11—C12—C13	−1.3 (4)
C1—C2—C7—C8	2.40 (19)	C11—C12—C13—C14	0.4 (3)
C3—C2—C7—C8	−113.79 (16)	C15—N1—C14—C13	−178.2 (2)
C1—C2—C7—C6	118.36 (16)	C15—N1—C14—C9	0.44 (19)
C3—C2—C7—C6	2.16 (17)	C12—C13—C14—N1	179.8 (2)
O3—C6—C7—C8	73.7 (2)	C12—C13—C14—C9	1.3 (3)
C5—C6—C7—C8	−177.5 (2)	C10—C9—C14—N1	179.13 (16)
O3—C6—C7—C2	−37.14 (17)	N2—C9—C14—N1	−0.36 (19)
C5—C6—C7—C2	71.6 (2)	C10—C9—C14—C13	−2.0 (3)
C1—N3—C8—O1	−177.7 (2)	N2—C9—C14—C13	178.48 (17)

### Hydrogen-bond geometry (Å, °)

**Table:**

*D*—H···*A*	*D*—H	H···*A*	*D*···*A*	*D*—H···*A*
N1—H1A···N2^i^	0.86	1.97	2.827 (2)	175

Symmetry codes: (i) *x*+1/4, −*y*+1/4, *z*+1/4.
